# Oral vascular leiomyoma with extensive calcification areas

**DOI:** 10.1590/S1808-86942010000400022

**Published:** 2015-10-19

**Authors:** Cassiano Francisco Weege Nonaka, Karuza Maria Alves Pereira, Márcia Cristina da Costa Miguel

**Affiliations:** a MSc in Oral Pathology. PhD Student - Graduate program in oral pathology - Federal University of Rio Grande do Norte; b MSc in Oral Pathology. PhD Student - Graduate program in oral pathology - Federal University of Rio Grande do Norte; c PhD in oral Pathology. Adjunct Professor - Graduate program in oral pathology - Federal University of Rio Grande do Norte

**Keywords:** mouth, diagnosis, leiomyoma, tongue

## INTRODUCTION

Leiomyomas are benign neoplasias originating from the smooth muscle tissue which rarely affects the oral cavity, with frequencies varying between 0.016% and 0.065%^1^, ^2^, ^3^, ^4^. There are less than 150 cases of oral leiomyomas reported in the literature so far1,4. Among the main histopathology of the oral cavity, we stress: solid leiomyomas, vascular leiomyomas and epithelioid leiomyomas^1^, ^2^, ^3^, ^4^.

## CASE REPORT

A 38 year-old man was referred to the Department of dentistry because of a painless lesion in his oral cavity, which had been there for about one year. Upon intraoral exam, we noticed an exophytic, nodular, red, well outlined lesion measuring about 2cm in diameter, located on the midline of the tongue dorsum.

There was no tongue mobility change or regional lymph node swelling and the patient's clinical history was uneventful. Given the clinical manifestations, there was the diagnostic hypothesis of a benign mesenchymal neoplasia, and we did a lesion excision biopsy.

**Figure 1:**
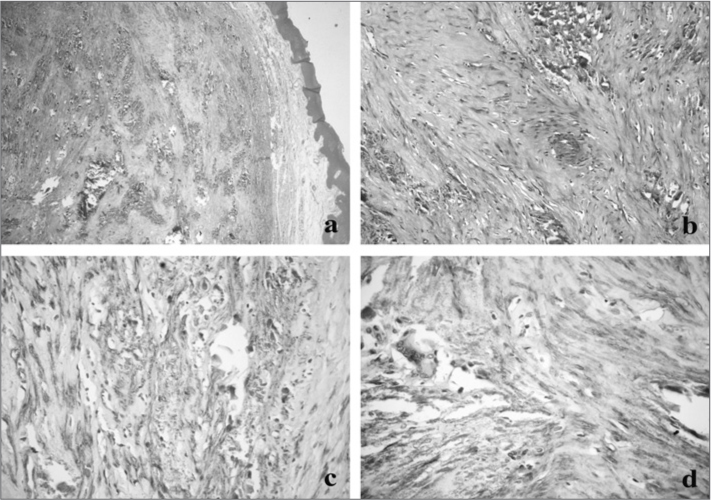
(a) Lesion separated from the oral mucosal coating by a band of fibrous connective tissue, showing extensive areas of calcification (HE/ 40x); (b) details of the calcification areas spread among the bundles of spindle-shaped cells (HE/ 200x); (c) intense marking for smooth muscle actin in the cytoplasm of neoplastic cells, interspersed by irregular calcifications (streptoavidin-biotin/ 400x); (d) presence of a multinucleated gigantic cell in association with calcification areas (streptoavidin-biotin/ 400x).

The material removed was referred to the Oral Pathology Lab. The microscopic analysis showed a fragment of benign neoplasia characterized by the proliferation of spindle-shaped nucleus cells, of blunt stumps, arranged in organized bundles on a straw-like shape or in a concentric pattern to numerous blood vessels. Amidst the specimen, we found extensive areas of calcification, of gross granules, or forming compact structures, located on the vascular lumen and spread in the stroma (Figures 1a and 1b).

Given the unusual histopathology for oral cavities leiomyomas, for diagnostic confirmation purposes, we did an immunohistochemical analysis, which revealed an intense cell staining for smooth muscle actin (SMA) (Figures 1c and 1d) and negativity for the S-100 protein, confirming the suspicion of a muscle origin. Thus, the lesion was diagnosed as vascular leiomyoma with intense calcification.

The patient continues under periodic observation and after one year and three months of the surgical intervention there were no traces of lesion recurrence.

## DISCUSSION

Oral cavity leiomyomas are uncommon lesions, representing only 0.016% to 0.065% of all the leiomyomas^1^^,^^3^^,^^4^. In the oral cavity, the vascular leiomyomas are the most common subtypes, making up about 64.0% − 74.0% of all the leiomyomas in this location3,4. It is likely that the higher frequency of this variant is associated with the main source of smooth muscle in the oral cavity, represented by the wall of blood vessels^3^.

There is no predilection for gender and most of the cases are diagnosed in patients aged between 40 and 59 years^1^, ^2^, ^3^. The lips represent the main anatomical site of these lesions (48.6% of the cases), with only 9.2% of the cases located on the tongue^1^.

Most of the vascular leiomyomas are nodular, painless and slow growth lesions, with less than 2 cm in diameter and a color which can vary between white to blue^1^^,^^2^. Such clinical presentation may mimic different other lesions, such as benign mesenchymal tumors, salivary gland lesions and vascular lesions^1^, ^2^, ^3^, ^4^.

For the ultimate diagnosis, other neoplasias made up of spindle cells must be ruled out, such as neurofibromas and neurilemomas^1^^,^^3^^,^^4^. In this context, the immunohistochemical technique is an important aid, in which these tumors express immunoreactivity for SMA and negativity for the S-100 protein^2^, ^3^, ^4^.

Calcification areas can be identified in some oral leiomyomas, however they are less expressive in histological findings^1^^,^^2^. Thus, the presence of extensive calcification areas makes the case very unique. These extensive calcification areas in leiomyomas are a topic for discussion, being considered from a tumoral dystrophic calcification process^5^ to unspecific degenerative changes^6^.

Regardless of the histopathology findings, vascular leiomyomas are treated by means of a surgical excision and recurrences are rare^1^, ^2^, ^3^, ^4^, ^5^. Although they are well vascularized lesions, important hemorrhagic events during the exeresis of these lesions are not common^1^^,^^3^.

## FINAL REMARKS

The case hereby presented stands out because of the peculiarities of the histopathology findings, especially on the form of extensive calcification areas - one aspect which, so far, has not been reported concerning oral cavity leiomyomas. In this context, the use of immunohistochemistry proves to be an important helping tool.
